# Post‐COVID‐19 Condition in Track and Field Master Athletes: Severity, Symptoms, and Associations With Quality of Life and C‐Reactive Protein Levels

**DOI:** 10.1111/sms.70106

**Published:** 2025-07-12

**Authors:** Boyi Zhang, Marijke Grau, Christian Puta, Daniel Arvidsson, Michael Arz, Jonas Böcker, Philip Chilibeck, Scott C. Forbes, Claudia Kaiser‐Stolz, Natalie McLaurin, Eri Miyamoto‐Mikami, Dominik Pesta, Willi Pustowalow, Hirofumi Tanaka, Jörn Rittweger, Wilhelm Bloch

**Affiliations:** ^1^ Institute of Cardiology and Sports Medicine German Sport University Cologne Cologne Germany; ^2^ Department of Sports Medicine and Health Promotion Friedrich‐Schiller‐University Jena Jena Germany; ^3^ Department for Internal Medicine IV (Gastroenterology, Hepatology and Infectious Diseases) Jena University Hospital Jena Germany; ^4^ Center for Sepsis Control and Care (CSCC) Jena University Hospital/Friedrich‐Schiller‐University Jena Jena Germany; ^5^ Department of Food and Nutrition and Sport Science University of Gothenburg Gothenburg Sweden; ^6^ Institute of Aerospace Medicine German Aerospace Center (DLR) Cologne Germany; ^7^ College of Kinesiology University of Saskatchewan Saskatoon Canada; ^8^ Department of Physical Education Studies Brandon University Brandon Canada; ^9^ Department of Kinesiology and Health Education The University of Texas at Austin Austin USA; ^10^ Faculty of Health and Sports Science Juntendo University Inzai Japan; ^11^ Medical Faculty University of Cologne Cologne Germany; ^12^ Cologne Excellence Cluster on Cellular Stress Responses in Aging‐Associated Diseases (CECAD) University of Cologne Cologne Germany

**Keywords:** long COVID, masters athletes, post‐COVID‐19 condition syndrome, quality of life

## Abstract

Here, we assessed the prevalence of post‐COVID‐condition (PCC, also known as long‐COVID) and investigated its associations with health‐related quality of life and immune‐related biomarkers in track and field masters athletes (MAs). A total of 216 MAs (114 males, 102 females; age: 58.3 ± 11.9 vs. 56.6 ± 11.7 years; BMI: 23.6 [22.2–24.8] vs. 21.3 [20.0–23.6] kg/m^2^) reported their post‐COVID‐conditions via the Post‐COVID Syndrome Questionnaire (PCSQ). In a subgroup of 108 MAs, fasting blood samples were collected to assess C‐reactive protein (CRP) levels as a biomarker of immune status (MAs‐CRP). Based on their PCSQ sum score, MAs were divided into three groups: no/mild, moderate, and severe. Associations between PCC severity and sex, athletic specialty, and competition level were evaluated using Fisher's exact test. Forty‐six (21%) MAs were identified with clinically relevant moderate‐to‐severe post‐COVID‐19 conditions (PCSQ score > 10.75). The most frequently reported symptoms included musculoskeletal pain (15%), sleep disturbance (13%), sensory or respiratory symptoms (11%), fatigue (11%), and flu‐like symptoms (11%). PCC prevalence did not differ by sex, athletic specialties, training load, or prior competition level (all *p* > 0.05). MAs with moderate‐to‐severe PCC had significantly lower physical and mental component scores of quality of life compared with those with no or mild symptoms (*p* < 0.05). In the MAs‐CRP subgroup, self‐reported cardiac ailments and flu‐like symptoms were significantly and positively associated with CRP levels (Spearman *ρ* = 0.27–0.30, all *p* < 0.01). Post‐COVID‐19 condition is associated with reduced quality of life in track and field masters athletes, independent of sex, prior competition levels, and training characteristics. Furthermore, low‐grade inflammation based on CRP levels was associated with self‐reported cardiac and flu‐like symptoms.

## 1 Introduction

Since the global outbreak of the SARS‐CoV‐2 infection, increasing research has focused on its long‐term health effects, known as post‐COVID‐19 condition (PCC) or long COVID, an infection‐associated chronic condition that occurs after SARS‐CoV‐2 infection and is present for at least 3 months as a continuous, relapsing and remitting, or progressive disease state that affects one or more organ systems [1]. PCC presents with highly heterogeneous clinical manifestations, affecting multiple systems and including symptoms such as fatigue, dyspnea, cognitive impairment, musculoskeletal issues, sleep disturbances, and autonomic dysfunction [2, 3, 4]. These symptoms can persist for months or even years, significantly impacting health‐related quality of life (HRQoL) [4]. A recent systematic review and meta‐analysis [5] reported a global prevalence of PCC at 42% (95% CI: 40%–44%). Despite its high prevalence, the etiology of PCC remains unclear.

Existing research suggests that regular physical activity (PA) prior to infection may help reduce the risk of SARS‐CoV‐2 infection and alleviate PCC symptoms [6]. Higher levels of PA are associated with lower chronic inflammation, enhanced immune regulation, and improved cardiorespiratory resilience, which may contribute to better recovery from viral infections and a reduced burden of symptoms compared to sedentary individuals [7]. However, immune function can be hampered in elite athletes, in particular during the competition season [8]. Recent studies on younger athletes have shown that PCC can lead to exercise intolerance and postexertional malaise [9]. Yet its impact on elite master athletes (MAs) remains unclear, despite their distinct physiological demands and recovery mechanisms [10].

MAs in track and field are generally defined as athletes aged ≥ 35 years who continue to compete after a professional career, transition from other sports, return to competition after a period of inactivity, or take up systematic training in adulthood to enhance physical fitness and gain better HRQoL compared with sedentary peers [11, 12, 13]. Their long‐term exposure to high‐intensity exercise may induce dual adaptive effects on the immune system [8]. On one hand, moderate exercise in elite athletes is positively correlated with regulatory T cell density, indicating a potential role in enhancing immune tolerance [14, 15]. On the other hand, overtraining or extremely high‐load training and competition may be associated with immune suppression, or postexercise transient lymphopenia, which could increase the risk of delayed recovery from viral infections [16].

Between 27% [17] and 62% [18] of athletes experience prolonged recovery lasting more than 28 days after SARS‐CoV‐2 infection. Common postinfection complications, such as acute exercise intolerance, postexertional malaise, muscle weakness, cardiovascular dysfunction, and persistent fatigue, can significantly impact athletic performance and overall well‐being [19]. While extensive research has focused on collegiate and elite young athletes, there remains a critical knowledge gap regarding the prevalence, symptom patterns, and impact of PCC in MAs [20]. To fill this gap, we aimed to investigate the prevalence of PCC in MAs and explore potential relations with athletic specialties, previous competition level, and training characteristics. Additionally, we assessed the impact of PCC on the HRQoL in MAs and investigated the role of inflammation in PCC susceptibility by analyzing inflammatory markers (C‐reactive protein, CRP). We hypothesized that the prevalence and symptom presentation of PCC would significantly impact the subjective quality of life in MAs, with its severity closely linked to inflammatory status.

## 2 Methods

This cross‐sectional, observational study investigated the prevalence of PCC in MAs and its association with HRQoL, training characteristics, and inflammation biomarkers. This project is part of an ongoing 10‐year cohort study on track and field MAs [21, 22]. This sub‐study focuses on PCC outcomes, utilizing questionnaire data and blood samples. Data were collected during the 2024 World Masters Athletics (WMA) Championship in Gothenburg, Sweden. Athletes who competed in the championship were invited to participate in our study through official email notifications from the organizing committee and on‐site flyer distribution.

A total of 335 MAs were enrolled in the study, with 237 completing the Post‐COVID Syndrome Questionnaire (PCSQ), resulting in a 71% response rate. However, a total of 21 participants were excluded from the general sample due to age < 35 (*n* = 2), missing data (*n* = 9), or training experience < 1 year (*n* = 10), see Figure S1. Among the 118 MAs who participated in blood collection under fasting and resting conditions, 10 were excluded for incomplete data (*n* = 4) or training < 1 year (*n* = 6). The final analysis included 216 MAs (Table 1).

**TABLE 1:** Participant characteristics.

Characteristic	Total (*n* = 216)	Sex		Athletic specialties			Previous highest competition levels during youth		
		Male (*n* = 114)	Female (*n* = 102)	Sprint (*n* = 84)	Endurance (*n* = 81)	Strength and power (*n* = 51)	Regional (*n* = 117)	National (*n* = 61)	International (*n* = 38)
Age (years)	57.5 ± 11.8	58.3 ± 11.9	56.6 ± 11.7	56.0 ± 11.6	58.3 ± 11.9	58.7 ± 12.2	58.8 ± 12.7	57.0 ± 10.7	54.3 ± 10.3
Height (cm)	170.5 ± 9.2	175.8 ± 7.5**	164.6 ± 7.1	171.2 ± 8.4	168.8 ± 10.1	172.1 ± 8.8	170.0 ± 8.8	170.8 ± 10.0	171.6 ± 9.1
Weight (kg)	66.7 ± 11.2	72.9 ± 8.7**	59.7 ± 9.4	68.4 ± 10.3“	63.3 ± 11.1	69.1 ± 11.6“	65.9 ± 10.7	67.9 ± 11.9	66.9 ± 11.7
BMI (kg/m^2^)	22.7 (20.8–24.5)	23.6 (22.2–24.8)**	21.3 (20.0–23.6)	23.4 (22.0–24.4)“	21.4 (20.1–23.7)	22.9 (20.4–25.0)	22.6 (20.9–24.3)	22.8 (20.6–24.8)	22.7 (20.5–23.8)
Training experience (years)	19.0 (7.4–30.1)	20.5 (9.3–39.0)*	17.0 (5.3–27.0)	16.0 (7.9–29.3)	14.0 (6.0–28.0)	24.8 (12.5–39.0)	16.0 (6.0–29.0)	20.0 (10.0–30.0)	26.0 (10.3–38.1)
Training hours (h/week)	11.0 (7.3–15.3)	10.7 (6.9–14.5)	11.1 (7.6–15.5)	10.7 (7.6–14.2)	13.3 (8.5–16.5)	7.6 (5.2–12.5)“	10.2 (7.3–14.3)	10.0 (6.3–15.3)	13.5 (10.7–18.2)^##,&^
Training RPE (AU)	17.0 (15.0–18.0)	17.0 (15.0–18.0)	17.0 (15.0–18.0)	17.0 (15.0–18.0)	17.0 (15.0–18.0)	15.0 (14.0–17.0)^^,‘	17.0 (15.0–18.0)	16.0 (15.0–17.0)	17.0 (15.0–18.0)
PCSQ score	0.0 (0.0–7.0)	0.0 (0.0–6.5)	0.0 (0.0–9.6)	0.0 (0.0–9.0)	0.0 (0.0–5.0)	0.0 (0.0–11.5)	0.0 (0.0–6.5)	0.0 (0.0–9.0)	0.0 (0.0–11.9)

*Note:* Parametric data such as age, height, and weight are presented as mean ± SD, while nonparametric data such as BMI, training years, training hours, RPE, and PCSQ scores are reported as median (25th–75th percentiles). Significant differences were determined using the Mann–Whitney test for sex comparisons, the Kruskal–Wallis test for athletic specialties and previous highest competition level, and Fisher's exact test for education level. Significance levels are denoted *p < 0.05, **p < 0.01, compared with females; ‘*p* < 0.05, “*p* < 0.01, compared with endurance athletes; ^*p* < 0.05, ^^*p* < 0.01, compared with sprint athletes. #*p* < 0.05, ##*p* < 0.01, compared with regional MAs; ^&^ *p* < 0.05, ^&&^ *p* < 0.01, compared with national MAs.

Abbreviations: BMI: Body Mass Index; PCSQ: Post‐COVID Syndrome Questionnaire; RPE: Maximal rating of perceived exertion in training (6–20 scale); Training hours/w: Average weekly track‐and‐field training hours.

All study procedures complied with the latest 2013 amendment of the Declaration of Helsinki and were approved by the Swedish Ethical Review Authority (Ref. No. 2023‐08100‐01) and the Ethical Committee of the Medical Chamber North‐Rhine (Ref. No. 2020401), Germany. All participants provided informed consent to participate in the study.

### 2.1 Measurements

Participants provided demographic information, including age, sex, educational background, previous competition level, athletic specialties, and current training characteristics via survey. The previous highest competition level was determined by the highest level of competition reached in any sport during their youth before participating in the Masters Athletic Championship (regional, national, and international). Athletic specialties were classified as follows: Sprint events include 100 m, 200 m, 400 m, and short‐distance hurdles. Endurance events include 5 km, 10 km, 20 km, marathon, steeplechase, 8 km cross‐country, walking events, 1500 m, and 800 m. Strength and power events cover shot put, hammer throw, weight throw, javelin, discus, throw pentathlon, long jump, high jump, triple jump, and pole vault. Athletes competing in multiple event categories were classified based on their best event, as indicated in the questionnaire. Training characteristics were evaluated using self‐reported retrospective average weekly training hours. Training intensity was measured with Borg's 6–20 rating of perceived exertion (RPE). Body mass index (BMI) was calculated using the formula (BMI = weight [kg]/height^2^ [m^2^]), measures obtained on‐site measurements taken with a wall‐mounted stadiometer and a digital scale.

### 2.2 Post‐COVID‐19 Condition

The Post‐COVID Syndrome Questionnaire (PCSQ) was used to assess the symptoms and severity of PCC through 12 binary (yes/no) items of known post‐COVID‐19 symptoms [23]. As the questionnaire specifically assesses symptoms in individuals with confirmed or probable COVID‐19 infection, MAs who reported no history of infection were instructed to skip it. Symptom weights were determined via regression analysis and integrated into a scoring system developed using k‐means clustering and ordinal logistic regression. The final PCSQ score is calculated by summing the weighted values, categorizing individuals into no/mild PCC (score < 10.75), moderate PCC (10.75–26.25), or severe PCC (score > 26.25, indicating clinically relevant PCC). The questionnaire has previously demonstrated excellent performance in distinguishing PCC severity (AUC = 0.996) [23] and exhibited good internal reliability in this study (Cronbach's *α* = 0.75). According to a previous study [24], analyzing PCC symptom clusters offers deeper insights into the factors contributing to PCC severity. Based on clinical relevance, we grouped these 12 symptoms into 8 clusters: fatigue (general fatigue and reduced exercise capacity), cognitive function (brain fog or similar issues), musculoskeletal system (joint and bone pain), sleep disorders (sleep disturbances), cardiac ailments (chest pain), gastrointestinal symptoms (digestive issues), flu‐like symptoms (ear, nose and throat (ENT) ailments like sore throat and runny nose, along with infection‐related symptoms), and other symptoms (taste or smell impairments, respiratory issues such as coughing and breathing difficulties, and skin problems like hair loss or rashes).

### 2.3 Health‐Related Quality of Life (HRQoL) Assessment

HRQoL was assessed using the 12‐item Short‐Form Health Survey (SF‐12) [25, 26], a widely used and validated tool with demonstrated reliability across athletic populations [27] and diverse international cultural settings [28]. The questionnaire evaluates eight domains, summarized into two composite scores: the physical component summary (PCS), which encompasses physical functioning, role limitations due to physical health, bodily pain, and general health, and the mental component summary (MCS), which includes vitality, social functioning, role limitations due to emotional problems, and mental health. These scores are calculated using standardized scoring algorithms and referenced against United States (US) population norms, where a value above 50 represents the average score for the US general population. Norm‐based scoring is preferred over the alternative 0–100 method, as it minimizes floor and ceiling effects and allows for meaningful comparisons across domains [29]. The Cronbach's *α* coefficient for SF‐12 in this study was 0.77, indicating good internal consistency.

### 2.4 Blood Samples and Immune Status

Venous blood samples (3 mL) were collected in the morning using serum separator tubes with clot activator gel. During blood test scheduling, participants were instructed to fast overnight, avoid intense exercise or competitions within 24 h, and refrain from coffee consumption prior to the blood draw. These instructions were provided verbally and reinforced through a written email reminder in the respective language of the participant to ensure compliance with testing guidelines. All samples were frozen after separation and shipped on dry ice for transportation to a certified medical laboratory in Cologne, Germany. Inflammatory status was assessed based on serum C‐reactive protein (CRP) concentrations. Quantitative CRP analysis was performed using an automated immunoassay analyzer (Atellica CH, Siemens; sensitivity: 0.5 mg/L), following the manufacturer's standardized protocols.

## 3 Statistical Analysis

Categorical variables were summarized as counts and percentages (%), while quantitative variables were expressed as means ± SD or medians (25th and 75th percentiles) for skewed distributions. Normality was assessed visually through histograms and verified using the Shapiro–Wilk test. Nonparametric analyses were applied to variables that did not meet normality assumptions. Between MAs with and without PCC, group comparisons of immune status, training volume (hours per week), training intensity (RPE), and SF‐12 physical and mental component summary scores were performed using Student's t‐test or the Mann–Whitney U test, as appropriate. For comparison among the three PCC severity groups, one‐way ANOVA with Tukey's post hoc test for normally distributed variables (e.g., age), while the Kruskal–Wallis test followed by Dwass–Steel–Critchlow–Fligner (DSCF) pairwise comparisons was applied to skewed continuous variables (e.g., BMI, training years, training hours, RPE, PCSQ scores, SF‐12 outcomes, and CRP levels). Fisher's exact test was used for categorical variables. Sex‐based comparisons were conducted within subgroups. Partial correlation analyses were performed to assess the relationship between the number of PCC symptoms and HRQoL subdomains. All analyses were conducted in R, with significance denoted as *p* < 0.05.

## 4 Results

### 4.1 Participants' Characteristics

A total of 216 MAs were included in the final analysis, comprising 114 males (52.8%) and 102 females (47.2%). Males were taller, heavier (all *p* < 0.01), and had more training experiences than females (*p* = 0.037, Table 1). Among athletic specialties, endurance MAs had significantly lower body weight and BMI compared with sprint and strength/power MAs (all *p* < 0.001, respectively), and trained significantly more hours per week than strength/power MAs (13.3 vs. 7.6 h·week^−1^, *p* = 0.001). Training intensity was higher in both endurance and sprint MAs compared with strength/power athletes (*p* = 0.001, Table 1). When analyzed by previous competition level during their youth, MAs who previously competed at the international level had more training hours than those who previously competed at the national and regional levels (13.5 vs. 10.0 vs. 10.2 h·week^−1^, *p* = 0.01; Table 1).

### 4.2 Prevalence of PCC and Its Association With HRQoL

**FIGURE 1:**
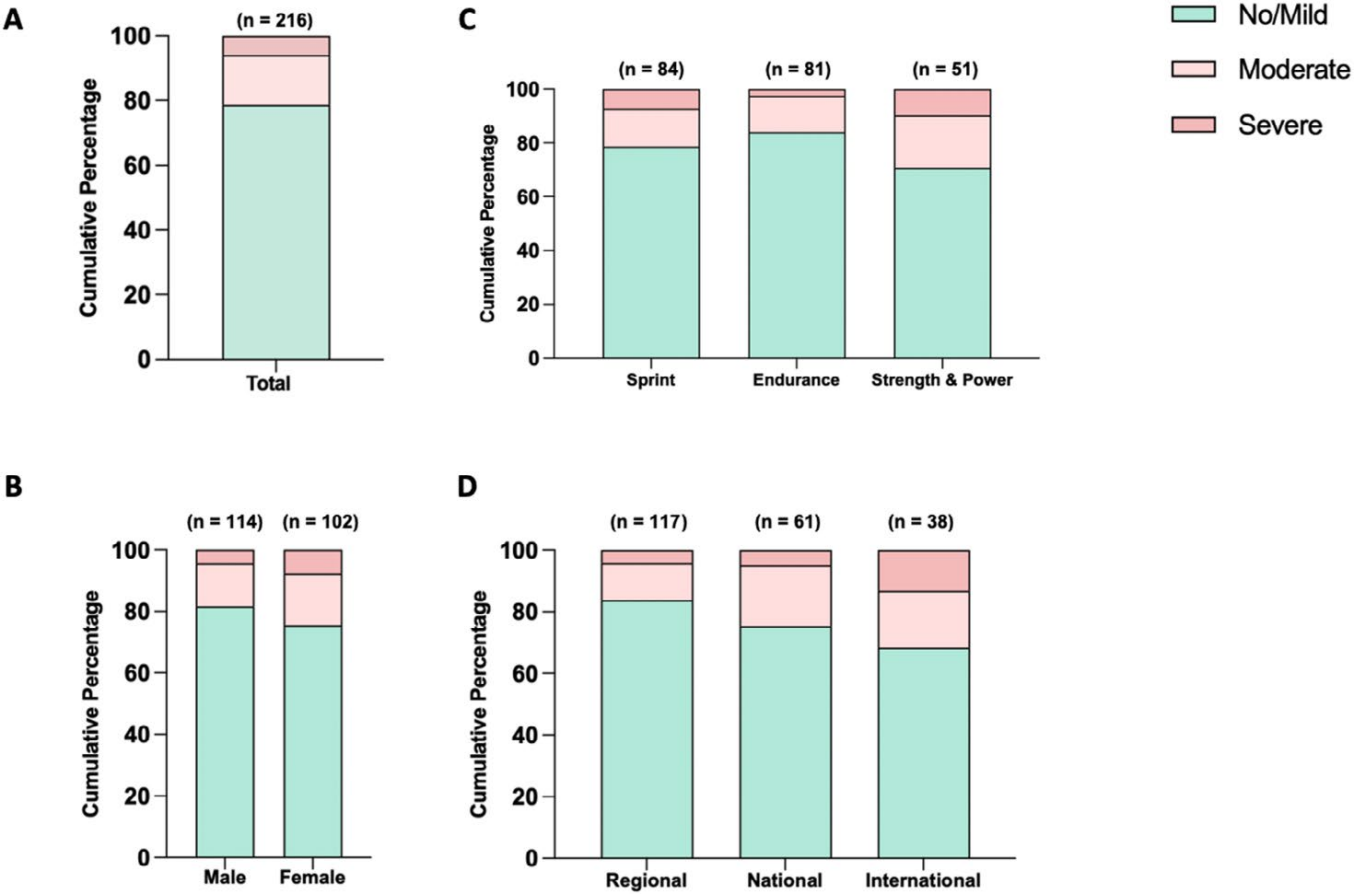
Post‐COVID‐19 condition (PCC) prevalence rate of masters athletes across sex, athletic specialties, and previous highest competition levels. (A) Overall prevalence in the study sample. (B) Prevalence stratified by sex. (C) Prevalence across athletic specialties. (D) Prevalence by previous highest competition level. Data are presented as cumulative percentages.

PCSQ scores differed significantly across athletic specialties (*p* = 0.045); however, DSCF post hoc pairwise comparisons showed that although strength/power MAs and sprinters tended to have higher scores than endurance MAs, these differences did not reach statistical significance (*p* = 0.081 and *p* = 0.083, respectively). In total, 21% (*n* = 46) of MAs were classified as having PCC, with 15% (*n* = 33) experiencing moderate, and 6% (*n* = 13) severe PCC (Figure 1A). PCC prevalence was not significantly associated with age, BMI, training years, weekly training hours, or RPE (all *p* > 0.05; Table 2). No significant differences in PCC prevalence were found across sex (*p* = 0.46; Figure 1B) and athletic specialties (*p* = 0.31), although strength and power MAs were more frequently classified with moderate (20%) or severe (10%) PCC compared with the other two specialties (Figure 1C). Despite a trend for a higher PCC prevalence (32%) among MAs competing at the international level during their youth than the other two groups (25% for national level and 16% for regional level), previous competition history was not significantly associated with PCC severity (Figure 1D, *p* = 0.152).

**TABLE 2:** Correlations between the number of PCC symptoms and HRQoL domains and subscales.

Variables	Spearman's *ρ*	*p*
SF‐12 PCS	−0.19	0.008**
Physical functioning	−0.14	0.04*
Role limitations due to physical health	−0.19	0.006**
Bodily pain	−0.27	< 0.001***
General health	−0.21	0.002**
SF‐12 MCS	−0.23	< 0.001***
Vitality	−0.18	0.011*
Social functioning	−0.23	< 0.001***
Role limitations due to emotional problems	−0.29	< 0.001***
Mental health	−0.22	0.001**

*Note:* Partial correlations are adjusted for age, BMI, training years, weekly training hours, training intensity (RPE), and athletic discipline. Significance levels are denoted as *p < 0.05, **p < 0.01, ***p < 0.001.

A significant and negative association was found between the number of PCC symptoms and HRQoL (Table 2). MAs with moderate‐to‐severe PCC had significantly lower PCS and MCS scores compared with those with no/mild PCC, indicating poorer HRQoL (*p* = 0.018 and *p* = 0.004, respectively). Additionally, symptom burden increased with PCC severity (*p* < 0.001), with median symptom cluster counts of 0 [0–1] for no/mild, 3 [2–3] for moderate, and 6 [5–6] for severe PCC (Table 3).

**TABLE 3:** Post‐COVID‐19 condition (PCC) severities among track and field MAs cohort.

Characteristic	PCC severities			*p*
	None/mild (*n* = 170)	Moderate (*n* = 33)	Severe (*n* = 13)	
Age (years)	57.5 ± 11.5	58.2 ± 13.0	56.2 ± 13.8	0.90
Female, *n* (%)	77 (45)	17 (52)	8 (62)	0.46
BMI (kg/m^2^)	22.8 (20.9–24.5)	21.7 (20.6–24.7)	23.0 (22.1–24.5)	0.82
Training experience (years)	16.0 (6.0–30.0)	21.0 (11.0–36.0)	21.0 (14.0–28.0)	0.20
Training hours (h/week)	11.0 (7.4–14.7)	11.0 (7.5–15.9)	8.0 (6.5–16.5)	0.69
Training RPE (AU)	17.0 (15.0–18.0)	17.0 (15.0–18.0)	17.0 (15.0–17.0)	0.96
SF‐12 PCS	55.2 (52.8–56.8)	51.9 (48.2–56.8)	52.4 (48.2–56.1)	0.05
SF‐12 MCS	62.2 (57.0–65.2)	59.1 (52.1–63.5)	56.5 (52.3–63.0)^a^	0.01
Number of symptoms	0.0 (0.0–1.0)	3.0 (2.0–3.0)^a^	6.0 (5.0–6.0)^b^	< 0.001
PCSQ score	0.0 (0.0–3.5)	13.5 (12.0–17.0)^a^	31.0 (27.5–35.5)^b^	< 0.001
Athletic specialties, *n* (%)				0.31
Sprint	66 (39)	12 (36)	6 (46)	
Endurance	68 (40)	11 (33)	2 (16)	
Strength and Power	36 (21)	10 (31)	5 (38)	

*Note:* PCC, Post‐COVID‐19 condition; Severity classification: None/Mild (PCSQ score ≤ 10.75), Moderate (10.75 < PCSQ score ≤ 26.25), Severe (PCSQ score > 26.25). SF‐12 PCS and SF‐12 MCS are the two composite scores (physical component score and mental component score) representing the participants' subjective physical and mental ability. Group differences were assessed using one‐way ANOVA for age (normally distributed), and the Kruskal–Wallis test followed by Dwass–Steel–Critchlow–Fligner (DSCF) pairwise comparisons for non‐normally distributed continuous variables. Fisher's exact test was used for categorical variables. ^a^Significantly different from none/mild group (*p* < 0.05); ^b^Significantly different from both none/mild and moderate groups (*p* < 0.001).

### 4.3 PCC Symptom Distribution: Moderate‐to‐Severe PCC MAs Have a Number of Multisystemic Symptoms

**FIGURE 2:**
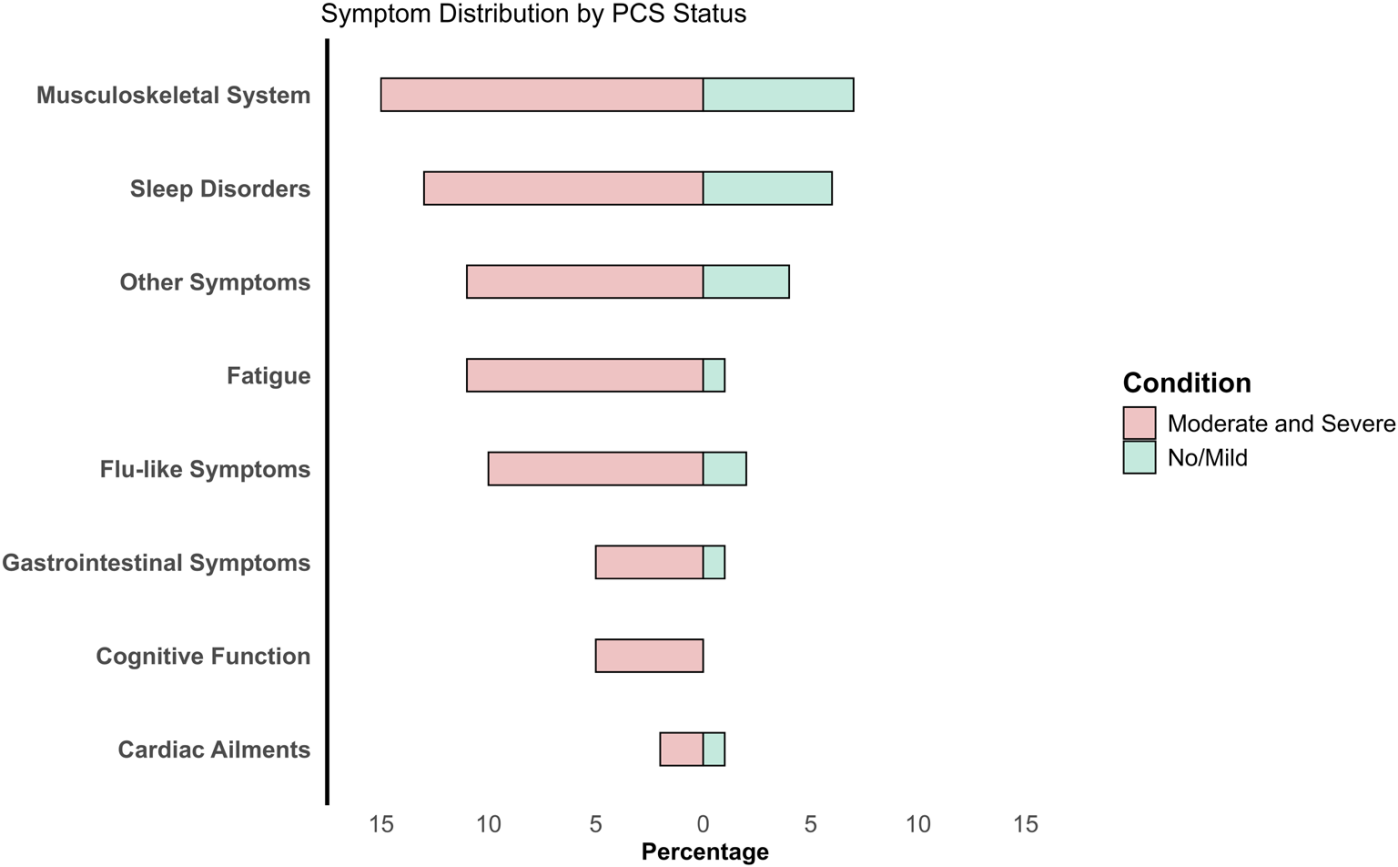
Symptom clusters in masters athletes with and without PCC. The bar graph presents the frequency of symptoms reported by MAs with No/Mild PCC (green) and moderate‐to‐severe PCC (red), ranked by prevalence in the moderate‐to‐severe PCC group. “Other symptoms” include taste or smell impairments, respiratory issues (e.g., breathing difficulties), and skin‐related conditions (e.g., hair loss or rashes).

MAs reported their most troubling symptoms experienced over the course of their infection. Figure 2 presents the symptom distribution across different PCC severities. Given the small number of severe PCC cases (*n* = 13), we combined moderate and severe PCC into a single group for illustration and analysis. The most frequently reported symptoms in MAs were musculoskeletal pain (15% vs. 7% in no/mild PCC), sleep disorders (13% vs. 6%), and other symptoms (11% vs. 4%), including taste or smell impairments, respiratory issues (e.g., breathing difficulties), and skin conditions (e.g., hair loss or rashes). Fatigue (11% vs. 1%) and flu‐like symptoms (10% vs. 2%), gastrointestinal issues (5% vs. 1%), and cardiac ailments (2% vs. 1%) were less common but remained more prevalent in moderate‐to‐severe PCC MAs. Notably, cognitive dysfunction (5%) was exclusive to the moderate‐to‐severe PCC group.

### 4.4 Subgroups Analysis by CRP and Its Association With PCC Symptoms Distribution

**FIGURE 3:**
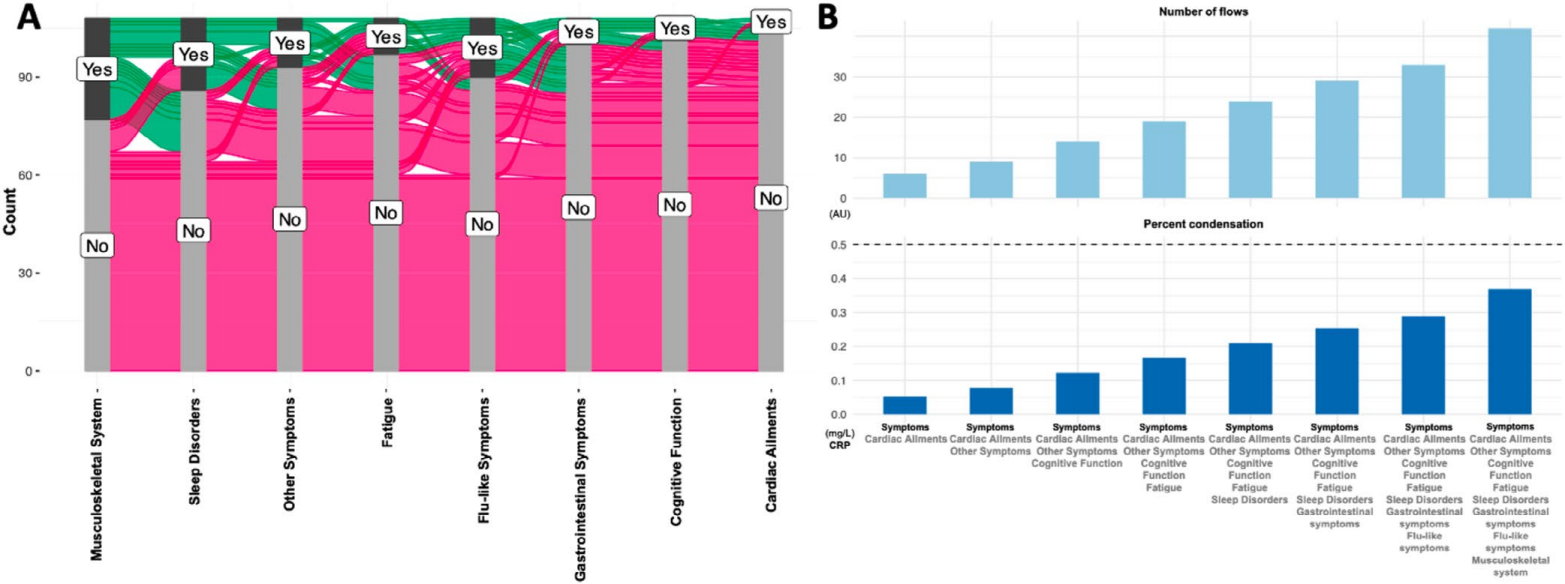
Multisymptom distribution among subgroups and its correlation with CRP levels. Co‐occurrence of long COVID‐19 symptoms visualized through an alluvial diagram (A) and a condensation plot (B). (A) The alluvial diagram represents the distribution of symptoms, with green indicating the presence and pink indicating the absence of symptoms across subgroups. (B) The condensation plot quantifies the number of unique symptom flows (top) and the percent condensation of CRP levels (bottom). Higher condensation percentages suggest that CRP is a key biomarker associated with symptom clustering. The dashed line in the lower plot represents the 50% condensation threshold. CRP, C‐reactive protein.

A subgroup analysis was conducted on MAs who participated in the blood draw (*n* = 108, Table S1). Among MAs with moderate‐to‐severe symptoms, males had higher, yet non‐significant, CRP levels than females (0.5 [0.5–1.2] vs. 0.5 [0.5–0.6] mg/l, *p* = 0.06). Further correlation analyses revealed significant positive associations between CRP levels and two PCC symptom clusters—cardiac‐related symptoms (Spearman's *ρ* = 0.27, *p* < 0.01) and flu‐like symptoms (Spearman's *ρ* = 0.30, *p* < 0.01; Figure S2). Additionally, we identified 27 distinct symptom distribution patterns, as visualized in the alluvial diagram (Figure 3A), providing a dynamic overview of how symptoms coexisted among MAs. The most common symptom patterns included musculoskeletal issues, sleep disorders, and various other symptoms such as taste or smell impairments, respiratory difficulties, and skin conditions like hair loss or rashes. An increasing trend in CRP levels was observed with the number of symptoms (Figure 3B).

## 5 Discussion

This study provides the first in‐depth analysis of PCC prevalence, symptom patterns, and their relations with HRQoL in MAs from different athletic specialties and previous competition levels. PCC affected 21% of MAs, with the most common symptoms including musculoskeletal issues, sleep disorders, and other symptoms (e.g., taste or smell impairments, respiratory issues, and skin conditions). Athletes with moderate‐to‐severe PCC had significantly lower PCS/MCS scores than MAs with no/mild PCC, highlighting its burden on physical and mental health and inverse association with their HRQoL. A severity‐dependent increase in symptom burden reinforced the multi‐systemic nature of PCC [30]. Collectively, these findings emphasize the need for targeted rehabilitation and long‐term monitoring in masters athletic populations.

In the present study, the overall prevalence of PCC among MAs in track and field disciplines was 21%. To provide context, previous systematic reviews [3, 31, 32] have reported a wide range of PCC prevalence, from as low as 7.5% to as high as 74.5%, reflecting substantial heterogeneity across studies. These variations are largely attributable to differences in study design, geographic region, case definitions, sample characteristics, and the duration of follow‐up [31]. Although the prevalence observed in our cohort appears lower, this finding aligns with the broad variability reported in the literature and may be partially explained by the high levels of physical activity and/or fitness as protective factors against severe COVID‐19 outcomes [7, 10]. Lifestyle factors such as nutrition and sleep quality may also contribute to the immune response against infection [6]. This study is the first to specifically investigate PCC prevalence in a MAs population. A comparison with prevalence rates reported in other athlete cohorts (4%–17%) [19] suggests that MAs demonstrate a higher prevalence of PCC. This may stem from differences in study populations, as previous research has predominantly focused on student and elite young athletes. Although age is a well‐established predictor of PCC severity in the general population [33], our findings indicate that this relationship may be less pronounced in highly active individuals. Indeed, PCC prevalence did not significantly differ across different age groups within the MAs cohort. This aligns with findings from a study on master endurance athletes (long‐distance runners and cyclists), where age did not significantly impact return to sport or the number of PCC symptoms [34]. Given that younger elite athletes typically engage in more training hours, have better access to up‐to‐date information on nutrition and training science, and benefit from structured support systems, their lower PCC risk may stem from a combination of these factors rather than age alone [17, 35]. Conversely, in the general population, age‐related PCC effects may be partially attributable to sedentary behavior rather than aging itself [36, 37].

MAs with PCC had significantly lower physical and mental component scores in the HRQoL assessment, compared with MAs with mild or no symptoms. This finding aligns with previous studies in other populations [38, 39, 40, 41], reinforcing the notion that PCC can have a profound impact on overall health and functional capacity. Additionally, a significant inverse correlation was observed between the number of symptom burdens and both physical and mental health scores, highlighting the detrimental effects of PCC on athletic performance and well‐being. This impact is particularly critical for MAs, as psychological health plays a key role in sustaining long‐term sports participation and competitive performance [42]. Furthermore, our study identified a stepwise increase in PCC symptom burden with greater severity, supporting the concept that PCC manifests as a multi‐system condition, with a greater symptom burden correlating with more pronounced limitations in athletic function and quality of life [41]. Notably, in our symptom pattern analysis, cognitive impairment (5%) was exclusively observed in the moderate‐to‐severe PCC group, suggesting that it may serve as a potential marker of PCC severity. This further underscores the broader implications of PCC beyond physical health, extending into cognitive function, which may further compromise training recovery and athletic performance, particularly in MAs engaging in long‐term high‐intensity training.

When comparing different athletic specialties, our study found no significant differences in PCC symptom prevalence across various disciplines, consistent with the previous study [34] that endurance running, cycling, and multi‐sport disciplines did not differ significantly in terms of PCC symptoms or RTS outcomes. However, our study differs from these findings in one key aspect: we observed that strength/power athletes (e.g., throwing and jumping disciplines) had a higher proportion of moderate‐to‐severe PCC cases, with PCSQ scores significantly exceeding those of endurance and sprint athletes. This discrepancy may reflect the different physiological adaptations associated with different sports disciplines. Strength and power events involve high‐load, intermittent efforts that may trigger distinct neuromuscular and immune stress responses, such as increased oxidative stress and chronic inflammation [16], which could contribute to both the onset and prolonged recovery of PCC symptoms. In contrast, endurance MAs tend to exhibit greater antioxidant capacity and superior cardiopulmonary adaptation [43], which may help reduce postinfection inflammatory burden and facilitate faster recovery. These differences in physiological adaptation may partially explain variations in PCC manifestation across athletic disciplines, though further longitudinal research is needed to validate this hypothesis.

Further investigation into the relationship between PCC and chronic inflammation revealed that CRP levels increased with the burden of flu‐like and cardiac ailment symptoms, although absolute values remained within subclinical ranges. This finding supports the hypothesis that chronic low‐grade inflammation, distinct from acute severe inflammatory responses may play a role in PCC pathophysiology [44]. Despite aligning with evidence of persistent immune dysregulation in PCC (e.g., unresolved viral antigen persistence or endothelial dysfunction) [45], it is noteworthy that CRP levels in our study were far below thresholds typically associated with severe systemic inflammation. This observation reflects findings from other studies, which report that many PCC cases, despite severe symptoms, often show normal or mildly elevated conventional biomarkers (e.g., CRP, D‐dimer) [46]. These patterns suggest that standard inflammatory tests may fail to capture the unique biology of mild yet persistent PCC. Even with these limitations, the observed trend between CRP levels and symptom burden highlights its potential as a relative biomarker for monitoring PCC progression in athletes. However, given the complex, multi‐system nature of PCC and the frequent disconnect between symptom severity and routine biomarker levels, future research should adopt a multi‐modal approach. Combining CRP with novel inflammatory markers (e.g., TNF‐α, IL‐6, or autoantibodies) and functional immune assays could provide a more accurate picture of subclinical inflammatory activity [47]. Such an approach may improve early detection of at‐risk athletes and inform targeted interventions, such as immune‐modulating therapies or personalized activity plans, even in the absence of clear biomarker abnormalities [48]. Importantly, rehabilitation protocols for MAs should take a precautionary stance. Inflammation management (e.g., stress reduction and anti‐inflammatory nutrition) should be integrated with graded physical reconditioning [49], recognizing that conventional biomarkers may underestimate the biological burden of PCC in mild cases [50].

## 6 Strengths and Limitations

This study has several strengths. This is the first evaluation of PCC prevalence, symptom patterns, and its impact on HRQoL in MAs. The inclusion of CRP as an inflammatory biomarker offers novel insights into the physiological mechanisms underlying PCC and its symptom burden. Finally, data collection was conducted in a real‐world competitive setting of a world master athletic championship, enhancing the ecological validity of the findings and their applicability to real‐life conditions.

Despite these strengths, some limitations should be acknowledged. Although this study is embedded within a prospective cohort design, the current analysis is based on baseline data and is therefore cross‐sectional; as such, causality between PCC and its associated factors cannot be inferred. For instance, self‐selection bias may have occurred if athletes with severe PCC chose not to compete, potentially underestimating symptom severity. While blood sampling followed a standardized protocol (fasted, collected in the morning, pre‐competition rested), the lack of objective monitoring for physical activity or dietary intake prior to testing may have introduced variability in metabolic and inflammatory markers. Additionally, we did not systematically record the timing of each athlete's competition events relative to blood sampling, limiting our ability to fully account for acute physiological responses to exercise or recovery status, particularly regarding CRP levels. Future studies should consider integrating detailed competition schedules and objective activity monitoring to better control for these confounding factors. Moreover, the reliance on self‐reported data (via the PCSQ) poses risks of recall bias. The absence of validated infection status and vaccination history, both key modifiers of PCC risk and severity, limits our ability to adjust for these confounders. A further limitation is the lack of occupational data, which may have influenced SARS‐CoV‐2 exposure risk and should be considered in future research to enhance risk profiling and interpretation of PCC outcomes [51].

## 7 Conclusions

This study provides novel insights into the prevalence and multi‐systemic symptomatology of PCC in track and field MAs, demonstrating a significant association between PCC severity and HRQoL decline. The association between elevated CRP levels and PCC symptom burden suggests a role for low‐grade inflammation in its pathophysiology. Additionally, differences in PCC prevalence across athletic specialties point to potential sport‐specific physiological adaptations. These findings highlight the need for early screening, ongoing health monitoring, and personalized rehabilitation strategies to better support MAs in PCC.

## Author Contributions

B.Z., W.B., and J.R. conceived the study, interpreted the data, and contributed to manuscript drafting and revision. B.Z. performed the data analysis. J.B. managed the survey implementation in REDCap. B.Z., D.A., M.A., P.C., S.C.F., C.K.S., N.M., E.M.‐M., D.P., W.P., H.T., and J.R. collected data during the championship. All authors substantially contributed to the manuscript's review, drafting, final approval, and take responsibility for its accuracy and integrity.

## Conflicts of Interest

The authors declare no conflicts of interest.

## Supporting information


Data S1.

## Data Availability

The data that support the findings of this study are available from the corresponding author upon reasonable request.
