# Supplementary material for: Post‐COVID‐19 Condition in Track and Field Master Athletes: Severity, Symptoms, and Associations With Quality of Life and C‐Reactive Protein Levels
Source: Scand J Med Sci Sports. 2025 Jul 12;35(7):e70106. doi: 10.1111/sms.70106 (PMC12254908; doi:10.1111/sms.70106)
Supplement: Supplementary file 1 — Data S1. [file SMS-35-e70106-s001.docx]

**Supplementary File**

**Supplementary Table 1. Blood sample subgroup characteristics.**

|  | **Total**  (n = 108) | **Sex** | |
| --- | --- | --- | --- |
| **Characteristic** |  | Male  (n = 53) | Female  (n = 55) |
| **Age (years)** | 58.4 ± 12.5 | 59.6 ± 13.3 | 57.2 ± 11.7 |
| **Height (cm)** | 169.7 ± 8.8 | 175.2 ± 7.4 ** | 164.5 ± 6.7 |
| **Weight (kg)** | 64.9 ± 10.8 | 71.8 ± 9.0 ** | 58.3 ± 8.0 |
| **BMI, kg/m^2^** | 22.1 (20.6 - 24.1) | 23.5 (21.6 - 24.8) ** | 21.1 (19.8 - 22.6) |
| **Education, n (%)** |  |  |  |
| **Primary school** | 1 (0.9) | 0 (0.0) | 1 (1.8) |
| **Secondary school** | 18 (16.7) | 9 (17.0) | 9 (16.4) |
| **Undergraduate** | 38 (35.2) | 16 (30.2) | 22 (40.0) |
| **Postgraduate** | 51 (47.2) | 28 (52.8) | 23 (41.8) |
| **Athletic Specialties, n (%)** |  |  |  |
| **Sprint** | 33 (30.6) | 20 (37.7) | 13 (23.6) |
| **Endurance** | 51 (47.2) | 22 (41.5) | 29 (52.7) |
| **Strength and Power** | 24 (22.2) | 11 (20.8) | 13 (23.6) |
| **Highest competitions level at youth, n (%)** |  |  |  |
| **Regional** | 65 (60.2 | 28 (52.8) | 37 (67.3) |
| **National** | 26 (24.1 | 15 (28.3) | 11 (20.0) |
| **International** | 17 (15.7) | 10 (18.9) | 7 (12.7) |
| **Training experience (years)** | 18.5 (7.0 - 30.6) | 16.0 (7.0 - 31.0) | 20.0 (6.0 - 29.5) |
| **Training hours (hr/wk)** | 9.9 (7.0 - 14.6) | 10.6 (6.9 - 15.0) | 9.8 (7.5 - 14.3) |
| **Training RPE (AU)** | 16.0 (15.0 - 17.0) | 16.0 (15.0 - 17.0) | 16.0 (14.0 - 17.0) |
| **PCSQ score** | 0.0 (0.0 - 8.6) | 0.0 (0.0 - 9.0) | 0.0 (0.0 - 6.5) |
| **PCC Severities, n (%)** |  |  |  |
| **None/Mild** | 86 (75) | 43 (75) | 43 (75) |
| **Moderate** | 19 (17) | 10 (18) | 9 (16) |
| **Severe** | 9 (8) | 4 (7) | 5 (9) |
| **CRP level (mg/L)** | 0.5 (0.5 - 0.8) | 0.5 (0.5 – 1.2) | 0.5 (0.5 – 0.6) |

Notes: PCC, Post-COVID-19 condition; Severity classification: None/Mild (PCSQ score ≤ 10.75), Moderate (10.75 < PCSQ score ≤ 26.25), Severe (PCSQ score > 26.25). CRP, C-reactive protein; Significant differences were assessed using the Mann-Whitney test for sex comparisons in age, anthropometric measures, training characteristics, PCSQ score, and CRP level, while Fisher’s exact test was used for education level, athletic specialties, highest competition levels, and PCC severity. Significance levels are denoted as *P < 0.05 and **P < 0.01.

**
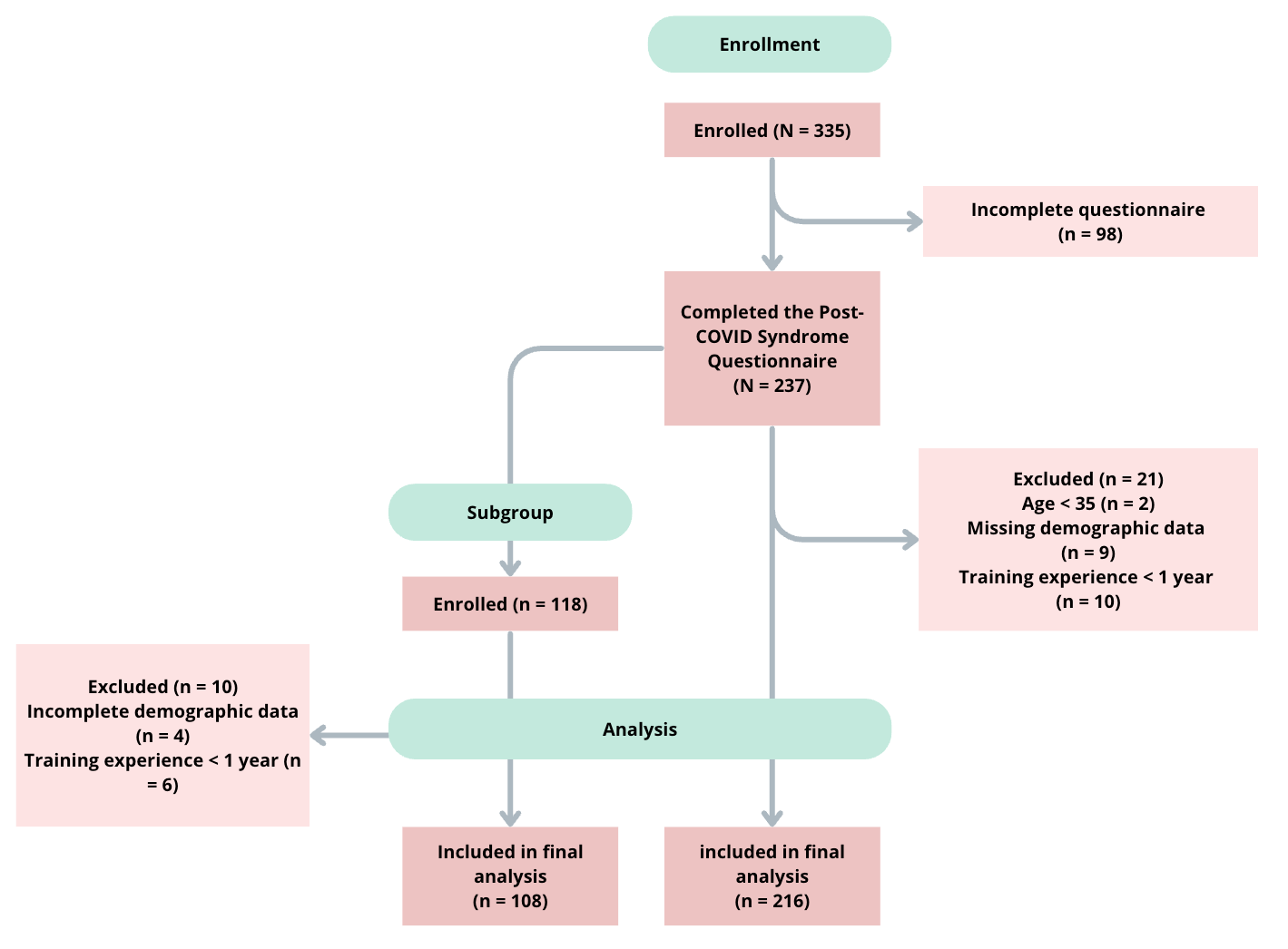
**

**Supplementary Figure 1. Flowchart of the study. A total of 335 master athletes (MAs) were enrolled in the study, of whom 237 (indicated in red) completed the Post-COVID Syndrome Questionnaire (PCSQ). Twenty-one participants were excluded due to age <35 years (n = 2), missing data (n = 9), or training experience <1 year (n = 10), resulting in 216 MAs included in the final analysis. In the blood sample subgroup, 108 MAs met the inclusion criteria and were analyzed.**


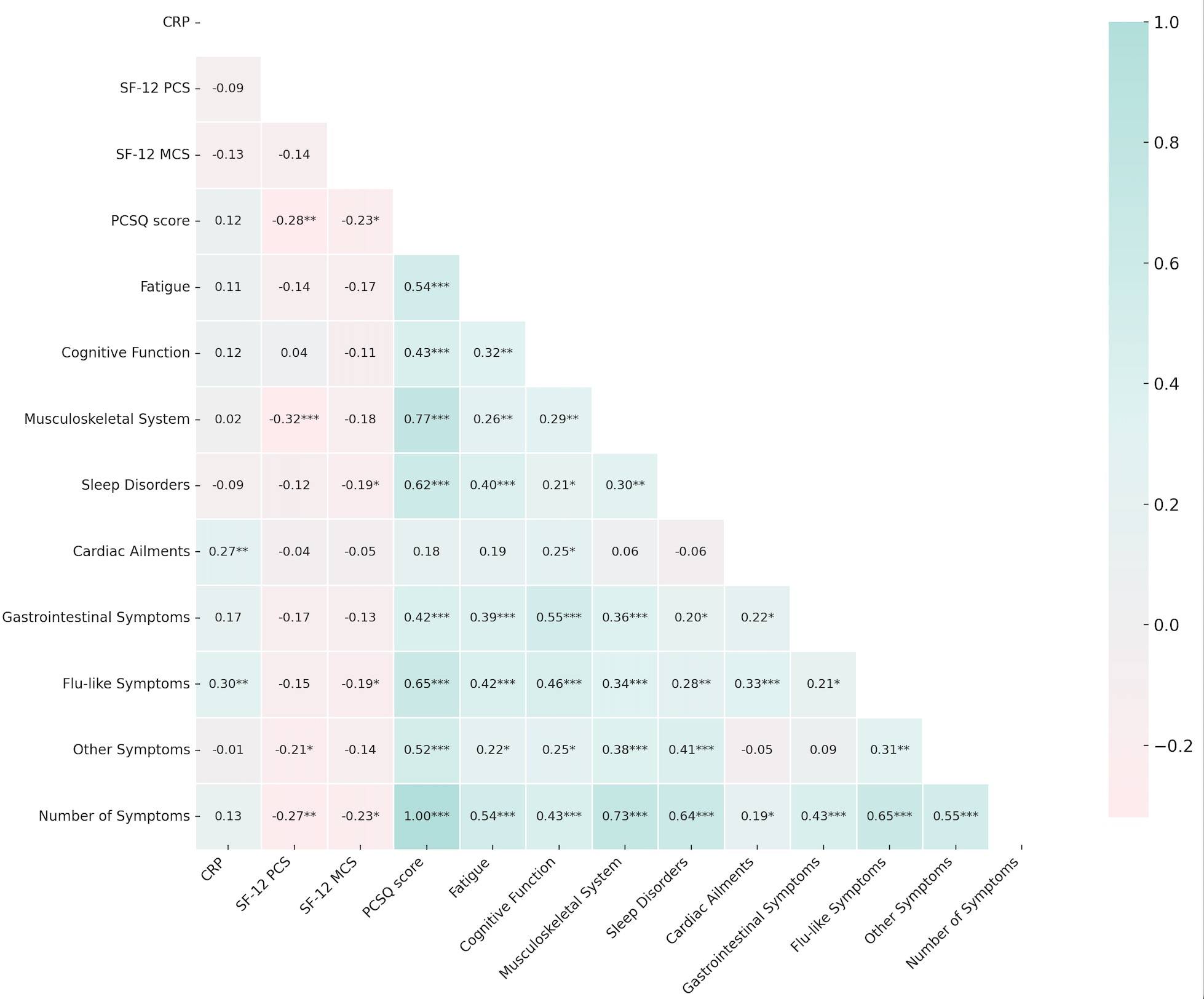


**Supplementary Figure 2. Partial Correlations between PCC symptoms and CRP levels.**

Note: Correlations were adjusted for age, BMI, training history (years), weekly training volume (hours and intensity), and athletic discipline. Significance levels: *P < 0.05, **P < 0.01, **P < 0.001.
